# Bullied at school, bullied at work: a prospective study

**DOI:** 10.1186/s40359-015-0092-1

**Published:** 2015-10-12

**Authors:** Lars Peter Andersen, Merete Labriola, Johan Hviid Andersen, Thomas Lund, Claus D. Hansen

**Affiliations:** 1 Danish Ramazzini Centre, Department of Occupational Medicine, University Research Clinic, Regional Hospital West Jutland, Gl. Landevej 61, 7400 Herning, Denmark; 2 Aarhus University, Department of Public Health, Universitets Parken, Aarhus, 8000 Denmark; 3 CFK Public Health and Quality Improvement Central Denmark Region, P. P. Orumsgade 11, Aarhus, 8000 Denmark; 4 Department of Sociology & Social Work, Kroghstrædet 5, Aalborg University, Aalborg, 9220 Denmark

**Keywords:** Adolescents, Bullying at school, Bullying at work, Risk factors, Prospective study

## Abstract

**Background:**

The consequences of childhood bullying victimisation are serious. Much previous research on risk factors for being bullied has used a cross-sectional design, impeding the possibility to draw conclusions on causality, and has not considered simultaneous effects of multiple risk factors. Paying closer attention to multiple risk factors for being bullying can provide a basis for designing intervention programmes to prevent or reduce bullying among children and adolescents.

**Methods:**

Risk factors for bullying were examined by using questionnaire data collected in 2004 and 2007. In 2004, the participants were aged 14–15 years and 17–18 years in 2007. The baseline questionnaire was answered by 3054 individuals in 2004, and 2181 individuals participated in both rounds. We analysed risk factors for being bullied at the individual and societal level. Information on the social background of the participants was derived from a national register at Statistics Denmark.

**Results:**

Several risk factors were identified. Being obese, low self-assessed position in school class, overprotective parents, low self-esteem, low sense of coherence and low socioeconomic status were risk factors for being bullied at school. Being overweight, smoking, low self-assessed position in class, low sense of coherence and low socioeconomic status were risk factors for being bullied at work. However, most associations between risk factors in 2004 and being bullied in 2007 disappeared after adjustment for being bullied in 2004.

**Conclusions:**

The strongest risk factor for being bullied was being previously bullied. Our results stress the importance of early prevention of bullying at schools. In addition, attention should be drawn to the role of overprotective parents.

## Background

Bullying has been conceptualised as a distinct type of aggression characterised by a multifaceted form of mistreatment seen mostly at schools and at work. The most widely employed definition of bullying emphasises persistent and repeated negative actions intended to intimidate or hurt a weaker person. Bullying includes acts of deliberate physical aggression (e.g. knocks, punches and kicks), verbal aggression (e.g. name calling and threats), relational aggression (e.g. social isolation and rumour spreading) and cyber aggression (e.g. text messaging and e-mailing hurtful messages or pictures) [1]. The negative interaction must occur relatively often (roughly on a weekly basis) and over a prolonged period of time (often 6 months) [2]. Given the seriousness of bullying, prevention of childhood and adolescent bullying has long been considered an important social and clinical problem.

### The prevalence of bullying

The prevalence of bullying among adolescents varies across countries. In two large cross-national studies – The Health Behavior in School-aged Children Survey and the Global School-based Students Health Survey – totalling 218,104 students in 66 countries aged between 11 and 15 years, the average prevalence of bullying victimisation at least once during the past month was 32.1 % in the first study, and in second study, 37.4 % of participants reported that they had been bullied at least once within the past 2 months. However, in both studies, a large variation in prevalence was found across countries, from 6 to 41 % in the first study and from 9 to 54 % in the second [3]. In spite of large differences in the prevalence of bullying, the results indicate that too many pupils suffer from being victims of aggressive acts intended to hurt them.

### The consequences of being bullied

The consequences of childhood bullying victimization are serious. Both cross-sectional and longitudinal studies have found that being a victim of bullying is associated with long-term psychological problems, including loneliness, general and social anxiety, diminished self-esteem, increased depressive symptoms and more frequent use of pain medication [4–6]. Finally, being a victim of bullying is an important risk factor for suicidal behaviour in adolescence and early adulthood [7, 8]. A review based on 37 studies found that any kind of participation in bullying increased the risk of suicidal ideation and/or behaviour. The strongest risk was for victim-perpetrators [7]. However, bullies also suffer because severe suicidal ideation has been found among both those who were bullied and among those who were bullies [9].

Not surprisingly, many antibullying programmes and interventions have been implemented in an attempt to reduce the prevalence of being bullied. Unfortunately, the success of intervention programmes to prevent or mitigate bullying in childhood and adolescence has been limited. A synthesis of the existing research on antibullying programmes concluded that the majority of programmes yielded no significant reductions in self-reported bullying, and therefore only cautious recommendations could be made [9].

### Theoretical frame of reference

Given the limited efficacy of bullying intervention programmes, the purpose of the present study was to more closely investigate multiple risk factors for bullying. Identifying risk factors can provide a basis for designing intervention programmes to prevent or reduce bullying among children and adolescents.

Studies on bullying at schools have identified several risk factors such as gender, age and deviate appearance of the victim; personal characteristics such as low self-esteem and lack of adequate coping skills; social status among peers and socioeconomic status in society. However, bullying is a complex phenomenon, and there is no single explanation for why some children are bullied by others [10]. Furthermore, bullying is conceptualised as a distinct type of aggressive behaviour, and psychological theories of aggression assert that the occurrence of aggression can seldom be reduced to one single factor but is more likely to be influenced by several factors simultaneously [11]. Aggressions, like other forms of complex behaviour, stem from the interplay of a wide range of personal, situational and social factors. Therefore, aggressive behaviour such as bullying occurs as a result of interactions between the persons involved and factors in the social context that may either facilitate or mitigate the risk of such behaviour [12, 13]. Although a detailed discussion of the causes of aggression is beyond the scope of this paper, it is important to underline the complexity of the task of identifying risk factors that are relevant in understanding bullying at schools and workplaces.

Several risk factors for being bullying at schools have been identified (for a review see [1]), but most studies identifying risk factors for bullying have included only a limited number of risk factors in their statistical models. All the risk variables may be correlated with each other, but some may be more important than others in predicting bullying. Therefore, the present study will simultaneously examine several different risk factors in young people related to their individual and personal levels, social levels and socioeconomic levels in order to identify the most important risk factors for bullying at schools and workplaces. This knowledge may be important in order to prevent bullying.

Preventing bullying may be good for the bullied as well as for the bullies in term of the negative psychological outcomes for both parties.

### Risk factors for bullying

Potential risk factors for bullying at the individual level include gender, age, physical appearance and health behaviour. Regarding gender, the results are inconsistent, and no substantial gender differences have been observed among adolescents in terms of the frequency of being bullied either at school or at work [14]. With respect to age, results from both cross-sectional and longitudinal studies on bullying at schools show that the prevalence of bullying tends to fall with age during adolescence [15–17].

When teens are asked why some adolescents are bullied, a common response is the deviant appearance of the victim [18]. Overweight and obesity have been found to be associated with an increased risk of being bullied in both cross-sectional studies and cohort studies [19–22]. Also, bullying has been found to be related to underweight in adolescents [23], and even short pupils are at greater risk of being bullied [24]. Apparently, any deviance from the physical norm may increase the risk of being bullied. Being a smoker also has been found to increase the risk of being bullied, but the results of studies on the relation between smoking and bullying are inconsistent [25–27].

At the personal level, self-esteem has been found to be associated with bullying [28]. Self-esteem refers to the global and evaluative view of oneself, and low self-esteem is associated with a variety of psychological dysfunctions, whereas high self-esteem is associated with social adeptness, leadership, higher levels of adjustment and good social skills. Therefore, because of poor social skills and low levels of adjustment, it seems plausible that low self-esteem may be a risk factor for being bullying. It is, however, unclear whether low self-esteem is a risk factor for bullying or a consequence of being bullied [29–31]. For instance, among 2326 Italian adolescents, Brighi et al. [30] found that low global self-esteem was a risk factor for victimisation, but on the other hand, another study found that victimisation was the most consistent predictor of low self-esteem [31]. Thus, it is unclear whether low self-esteem is a risk factor for or a consequence of being bullied. Furthermore, most studies are cross-sectional in design, making causal interpretations difficult. In this study, self-esteem was conceptualised as a risk factor, because it is considered to be a general internal presentation of social acceptance and rejection and a measure of social functioning. Thus, low self-esteem could be a risk factor for being a target of bullying.

Studies have demonstrated associations between an increased risk of being bullied and conflicts with parents and being from a family characterised by a punitive, conflicting and nonsupportive parenting style [10, 32]. Additionally, victims’ homes have been found to be characterised by a higher level of criticism and fewer rules [33] and having authoritative parents who rarely value their children and tend not to give them the opportunity to speak up for themselves [34]. Overly protective parenting style could be a risk factor for bullying as well because parents who are overly protective of their children and do not let them handle conflicts with peers by themselves may contribute to the causation of bullying [35, 36]. However, the causal direction is unclear because of the cross-sectional design of these studies, and protective parenting could also be an outcome of bullying.

One consequence of inadequate parenting style or poor family functioning may be children’s insufficient coping strategies. For instance, a study found a clear relation between perceived parenting practices and coping in offspring [37]. These researchers found that parenting characterised by warmth and acceptance involved both a high degree of parental monitoring but also parental demands for age-appropriate behaviour. Thus, the child may learn that events are to some degree controllable. The result of the study was that the children of parents using an accepting and warm parenting style more often used problem-focused coping strategies than did children who reported that their parents used other rearing styles. Therefore, one of the consequences of inadequate parenting style or poor family functioning may be inadequate coping skills and lack of social skills needed in order to resolve conflicts in the peer group or with work colleagues due to lack of experience with conflict resolution in the family. Based on this background, we sought to examine whether poor family functioning and overprotective parents predict bullying later at school or work.

The associations between bullying and coping have been examined in several studies, and the results show that victims of bullying lack adaptive coping strategies and more often use avoidant coping or similar strategies that might be considered similar [10, 38, 39]. For instance, one study found that victims of bullying rarely asked for help or talked about what happened, but instead remained passive [40]. Another study found a positive association between emotionally oriented coping strategies and victimisation [10]. Thus, it seems relevant to study the association between coping and bullying.

Regarding coping strategies, it is important to include the victims’ appraisal of the bullying situation because appraisals, according to the transactional theory of stress, determine the coping response, and thus victims’ perceptions of control become important for the implementation of coping strategies [41].

The concept of sense of coherence brings the perception of control and manageability into consideration. The concept refers to the individual’s perception of comprehensibility, meaningfulness and manageability, the last referring to expectations about the availability of adequate resources to cope with stressors. Sense of coherence affects how individuals perceive the events that happen to them, as well as the extent to which they perceive these events as controllable. Persons with a strong sense of coherence are described as more resistant to stress and able to cope adaptively [42], and studies have found a direct effect of sense of coherence on stress [43]. One study found that strong sense of coherence offered protective benefits to targets exposed to bullying [44], and another study found that employees with a low sense of coherence more often were subjected to violence [45]. In the present study, we analysed whether a low sense of coherence is a risk factor for bullying.

In regard to the social context, bullying is related to social status in the group. Indicators of status are social preference, popularity, school performance and socioeconomic status. Research has found that low levels of social preference and low levels of perceived popularity are associated with an increased risk of being bullied [46, 47]. However, the cross-sectional nature of the studies cannot exclude the possibility that low social status may be an outcome and not a precursor of bullying, and therefore more longitudinal studies are needed. Furthermore, a meta-analysis concluded that bullied pupils were more likely to achieve lower grades than nonbullied pupils. Low grades may reflect interpersonal and social difficulties that may increase the risk of bullying. However, the cross-sectional nature of the studies cannot exclude that bullying may lead to mental distress, which could affect school performance [48]. Although the negative relation between victimisation and academic performance is significant, there are few longitudinal studies on this topic. Thus, it is unclear whether victimisation can be conceptualised as a risk factor or an outcome of poor academic performance. In the present study, low social status and low school performance were conceptualised as risk factors for bullying; even though some longitudinal studies have shown that being a victim of bullying predicts later academic difficulties, there are only limited results supporting this notion [49].

Finally, the socioeconomic status in society is also related to bullying, and research has revealed that exposure to bullying is patterned by socioeconomic status because adolescents from lower socioeconomic status families are at higher risk of being bullied [50–53]

One explanation could be that inequalities in society may lead to more widespread approval of behaviours associated with social status differences such as bullying [54]. Furthermore, growing up in a low social status family might be associated with more stress in the form of unemployment, divorce, illness and moving, which might affect children’s adaptive skills [55], again possibly increasing the risk of being bullied.

### Stability of victimisation from bullying at school and at work

Given the negative consequences of bullying, it is important to examine the continuance continued risk of being bullied. In other words, is being bullied once a risk factor in itself for later victimisation?

Relatively little attention has been given to the effect of being bullied once on later victimisation, and the few studies that have examined the stability of victimisation from bullying during adolescence have found the risk of being bullied to be relatively stable. For instance, it was found that being a victim of bullying at age 8 was associated with victimisation 8 years later [8, 16]. So far only a few studies have differentiated between victimisation that continues from primary school to secondary school and from primary school to the workplace. Both at school and at work, environmental and organisational factors can be sources of bullying, so the stability of victimisation from primary school to the workplace would not necessarily be expected because environmental and organisational factors change. In spite of this, researchers have found that the highest risk factor for being bullied in the workplace was being a bully victim at school [56]. This relationship was seen for both males and females. Furthermore, another study found that victims who are bullied at school report being bullied at work somewhat more often than do others [57]. These results indicate that factors of continuity in the risk of victimisation could be more related to individual attributes such as low self-esteem, personality, lack of sufficient coping strategies and poor ability to establish protective social relationships than to environmental and organisational factors. While both studies were retrospective in design, which could have resulted in recall bias, the results indicate that being bullied once may be an important risk factor for being bullied later. In spite of the expansion of research on bullying victimisation, few studies have investigated possible links between individuals’ experience of previous victimisation at school and later victimisation at work or secondary school [58].

### Present study

Research has revealed several different risk factors for being bullied at school.

Using data from a prospective cohort study of young people from the western part of Denmark, this study examines several risk factors for being bullied at 17–18 years of age, making it possible to test the independent contribution of each risk factor after adjustment for covariates in the same domain.

The purpose of the present study was to identify risk factors measured at age 14–15 for being bullied at age 17–18 at either work or school. Additionally, the study examines the associations between victimisation at the age of 14–15 years and victimisation later at the age of 17–18 years at a site of higher education or at work.

More specifically, the purposes of the study were as follows:

1. To examine the prevalence of being bullied at age 15 and at age 18 at work and at school
2. To identity the most important risk factors for being bullied at age 18, including the following:
  - Individual risk factors: gender, body mass index, smoking, previously being bullied at age 15
  - Personal risk factors: self-esteem and sense of coherence at age 15
  - Social risk factors: parental relations and family function at age 15
  - Coping strategies: avoidance strategies and support seeking at age 15
  - Indicators of social status: social position in peer group and social position in society at age 15
3. To examine the continuity of being bullied from age 15 to age 18

## Methods

The data used in this study stem from the ongoing West Jutland Cohort Study (Vestliv), a birth cohort study following a complete regional cohort of adolescents born in 1989 in the county of Ringkoebing in the western part of Denmark. The cohort comprised 3681 individuals born in 1989, of which 3054 (83 %) answered the baseline questionnaire in 2004. Those who had not opted out of the study (N = 3293) were sent the second-round questionnaire in 2007, and 2400 (73 %) answered the questionnaire. In all, 2181 individuals participated in both rounds (59 % of the original cohort). These 2181 individuals constitute the primary study base, although some of the analyses were carried out on data collected in only one of the two rounds due to differences in the available variables.

The study gathered comprehensive information on the occurrence, severity and impact of manifold symptoms of physical and mental health problems, both self-reported and register-based [55]. In addition, information on exposures at school and at home was gathered. Finally, information on parental socioeconomic status was derived from national registers and linked to the questionnaire data.

Information on the social background of the participants (e.g. household income, parents’ highest education etc.) was derived from a national register at Statistics Denmark by using information from the Central Office of Civil Registration, in which the respondents are linked to their legal parents or guardians via a personal identification number given to everyone in Denmark at birth (or upon entry for immigrants). The study and the linking of information using the Central Office of Civil Registration were approved by the Danish Data Protection Agency (Study No. 2009-41-3761). The study was approved by the Danish Data Protection Agency and followed the regulations for data storage and protection

### Outcome measures

Bullying was measured using three single items. In 2004, the respondents were asked “How much have you been bullied at school during the last 6 months?” with the response categories “Never”, “Once or twice”, “A few times”, “Once a week” and “Several times a week”. For use in the analyses, this variable was recorded as “has ever been bullied” and “has not been bullied at all”. In 2007, two single measures were used with slightly different wording. The respondents were asked: “How much have you been bullied in an unpleasant way at school during the last 6 months?” with the same response categories as in 2004. In addition, the question was asked about bullying at work for the respondents who had a job at the time of the baseline questionnaire. 478 of those studied did not have a job, 1305 were at school and had a job, 348 were trainees and 125 had an ordinary job. Both questions on bullying in 2007 were recorded in the same way as the 2004 variable.

### Health-related lifestyle

Two indicators of health-related lifestyle, smoking and Body Mass Index were included as possible risk factors for being bullied. Daily smoking and overweight/obesity (defined according to the Body Mass Index-for-age values defined by World Health Organisation at age 15) were included in the analyses as indicators of unhealthy lifestyle that may be stigmatising and thus lead to a higher risk of being bullied.

### Indicators of status

Two traditional measures of parental socioeconomic status were derived from information about the participants’ parents from national registers on income and education in 2003 (i.e. the year before baseline data was collected). Household income and highest attained educational level in the household were used as measures of socioeconomic status. If the participants’ parents were divorced, information on the household in which the participants had their place of residence according to the Central Personal Register was used. Information on income was taken from the tax register (recoded into tertiles for some of the analyses), and information on educational attainment was taken from the Danish Educational Register and recoded into three categories: compulsory school (<10 years of education), high school/vocational training (10–12 years) and short, medium or higher education (>12 years).

Another mechanism hypothesised to be responsible for bullying is differences in the social position of adolescents in their peer groups foremost in their school class. In this study, The MacArthur Scale of Subjective Social Status – Youth Version was used [59].

### Parental relations

Parent overprotection was measured using four items from the short version of the Parental Bonding Instrument [60] (Cronbach’s alpha = 0.69). Another aspect of parental relations is summed up in the Family Functioning Index, which taps into how well conflicts are resolved in the family. Family functioning was measured using 12 items from the Family Assessment Device – General Functioning scale (FAD-GF) [61]. (Cronbach’s alpha = 0.86). Both indicators of parental relations were dichotomised for the multivariate analyses using the median as cut-off point.

### Personal psychological characteristics

Two different aspects of the adolescents’ personal characteristics were included in the analyses. First of all, self-esteem – measured by the 6-item version of Rosenberg’s global self-esteem scale [62] – was included (Cronbach’s alpha = 0.82). Secondly, the four items tapping into the meaningfulness dimension of Antonovsky’s construct SOS were also included [42] (Cronbach’s alpha = 0.62). One could argue that the measure of internal consistency was a little low. However, comparing our results with those of other translations, the consistency actually appeared to be somewhat higher in our sample [63]. Coping was measured using two scales based on the Brief COPE Scale used in a previous paper based on data from this cohort [55]. The original subscales were divided into two coping dimensions that emphasised either an “active” approach to problem solving, generally considered to be more adaptive, or “avoidance”-based approach, considered to be less adaptive. The six items from the subscales “active coping”, “planning” and “positive reframing” were combined to form the “active” coping scale (Cronbach’s alpha = 0.75), and the four items from the subscales “self-distraction” and “behavioural disengagement” were used to form the “avoidance” coping scale (Cronbach’s alpha = 0.53). Once again, one could argue that the measure of internal consistency was a little low, but our sample’s consistency appeared to be higher than that of other translations. Cronbach’s alphas for the coping subscales were similar to the alpha values reported by Carver, supporting the reliability of our measures despite the somewhat low alpha coefficients. Both scales were created using the mean of the items, thus yielding two scales with scores between 1 and 4, with higher scores indicating higher levels of that type of coping. All four measures of psychological characteristics were dichotomised for use in the multivariate analyses using the median as cut-off value.

### Statistical analyses

Table 1 shows all the characteristics of the study sample. Multiple logistic regression models were used to study the association between bullying and the two dependent variables. For use in the logistic regression models, all of the scales were dichotomised using the median score as the cut-off point, e.g. creating a variable coded 1 if the respondent had levels of self-esteem below the median. Four models were tested. The bivariate association was calculated between each of the independent variables and bullying (Model 1). After this procedure, the independent variables were entered into models by the abovementioned themes to test the independent contribution of each of the variables after taking into account other aspects of the same processes (Model 2). Bullying experience in 2004 was entered into the domain-specific models (Model 3). Last, a final model in which stepwise forward selection was used to reduce the number of independent variables was created (Model 4). The Hosmer-Lemeshow goodness-of-fit was used to evaluate the quality of the models [64]. All in all, the sample used for the multivariate analyses consisted of 1853 and 1376 respondents being bullied at school and at work, respectively; participants were excluded on a model-based basis, so the number of participants in each model varied according to the number of missing data elements on any of the variables. The analyses were performed using STATA 13 (Stata Statistical Software: Release 13. College Station, TX, USA: StataCorp LP).

**Table 1:** Characteristics of study population of children at age 15 years. N = 2,278

Variable name	Categories	N	Pct/mean (SD)
Gender	Female	1,237	54.3
	Male	1,041	45.7
*Lifestyle factors*			
Body Mass Index for age	Severe thinness	57	2.9
	Normal weight	1,640	83.0
	Overweight	216	10.9
	Obese	62	3.1
Smoking habits	Not smoker smoke	1,857	89.3
	Daily smoker	222	10.7
*Indicators of status*			
School performance	Mean (SD). scale; 0-13	8.99	1.17
Self-assessed position in school class	Mean (SD). scale;1-10	7.08	1.72
Parental educational level	<10 year	255	11.2
	10-12 years	1,169	51.4
	>13	852	37.4
Household income, DKR	Mean (SD)	2,276	573,574 (250,511)
*Social relations with parents*			
Overprotective parents	Mean (SD) scale; 4-16	8.10	2.63
Family functioning	Mean (SD), scale;1-4	1.72	0.50
*Personal psychological characteristics*			
Self-esteem	Mean (SD) scale: 6-24	19.08	2.90
Sense of Coherence (Meaningfulness)	Mean (SD) scale: 5-20	14.37	2.14
Active coping	Mean (SD) scale: 1-4	2.65	0.52
Avoidance coping	Mean (SD) scale: 1–3.5	1.94	0.47

## Results

Table 2 shows the prevalence of being bullied at age 14–15 at school and age 17–18 at school and at work. Due to changes in the wording of the question, it was not possible to compare the prevalence across years. At age 17–18, it appeared that bullying was more prevalent in the school context than at the adolescents’ workplaces. A very low number of individuals were exposed to weekly bullying behaviour; less than 10 % of those reporting any bullying were bullied weekly or more often.

**Table 2:** Prevalence of bullying at school and at work at ages 15 and 18

Variable	Level	N	Pct
Bullied at school during last 6 months (age 14–15)	Not bullied	2,255	74.6
	Bullied	766	25.4
	Frequently bullied^a^	76	9.9
Bullied in an unpleasant way at school during last 6 months (age 17–18)	Not bullied	1,843	87.8
	Bullied	256	12.2
	Frequently bullied^a^	12	4.7
Bullied in an unpleasant way at work during last 6 months (age 17–18)	Not bullied	1,535	91.4
	Bullied	144	8.6
	Frequently bullied^a^	8	5.5

^a^Frequently bullied = Bullied at least once a week/Ever bullied

In Table 3, the association between bullying at age 14–15 and bullying in either setting at age 17–18 is shown. The risk of experiencing bullying at school at age 17–18 was approximately twice as high for those who were bullied at school at age 14–15. The association was somewhat stronger for bullying at work. Nearly 45 % of those who experienced bullying at school at age 14–15 also experienced bullying in their higher educational track at age 17–18 or at work 3 years later.

**Table 3:** Association between bullying at age 15 and bullying at age 18. Percentages and prevalence proportion ratios (PPRs) with 95 % confidence intervals

	Proportion bullied at school (age 18)		Proportion bullied at work (age 18)	
	N (Pct)	PPR (95 % CI)	N/Pct	PPR
Not bullied at school age 15	69 (6.0 %)	1 (ref)	123 (8.5 %)	1 (ref)
Bullied at school at age 15	56 (15.1 %)	1.99 (1.56-2.54)	107 (23.4 %)	2.23 (1.86-2.67)

Table 4 displays risk factors for bullying experiences at school at age 17–18. Only three variables remained statistically significantly associated with bullying after mutually adjusting for each other and for being bullied at age 14–15. First of all, having been bullied at school at age 14–15 raises the risk of experiencing being bullying 3 years later by factor of 3 even when after taking other possible factors into account. Secondly, having a parent with a parenting style more overprotective than the median also raised the risk of being bullied. For those scoring in the highest decile on the scale, the risk of being bullied was more than twice as high as it is for those having the least overprotective parents. Many of the included variables were bivariately associated with bullying, e.g. Body Mass Index and measures of social position in the school class attended at age 14–15. These associations were diluted and largely disappeared after mutually adjusting for all the variables used, as well as after entering the variable indicating whether the respondents had experienced bullying at age 14–15.

**Table 4:** Associations between and lifestyle, personal characteristics, peer group and parental relations at age 15 and being bullied at school age 18. Odds ratios (OR) obtained by logistic regression with 95 % confidence intervals

		Model 1 Bivariate	Model 2^a^ Adjusted for covariates in same domain	Model 3^a^ Adjusted for covariates in same domain and bullying	Model 4** Forward stepwise selection
		OR (95% CI)	OR (95 % CI)	OR (95 % CI)	OR (95 % CI)
Gender					
Girls	Ref	1.00		1.00	1.00
Boys		1.36 (1.04-1.76)		1.37 (1.04-1.82)	1.40 (1.04-1.89)
Lifestyle factors					
Body Mass Index					
Underweight		1.18 (0.53-2.66)	1.18 (0.53-2.65)	1.17 (0.51-2.68)	removed
Normal weight	Ref	1.00	1.00	1.00	
Overweight		1.35 (0.88-2.06)	1.35 (0.89-2.07)	1.22 (0.79-1.89)	
Obesity		2.27 (1.17-4.41)	2.37 (1.17-4.40)	1.68 (0.85-3.33)	
Smoking					
Does not smoke	Ref	1.00	1.00	1.00	removed
Smoke		1.14 (0.73-1.77)	0.98 (0.46-2.08)	0.95 (0.44-2.06)	
Social position in peer group					
*School performance*					
School performance above median	Ref.	1.00	1.00	1.00	removed
School performance below median		1.26 (0.95-1.67)	1.06 (0.78-1.43)	1.04 (0.76-1.42)	
*Self-assessed position in schoolat school class*					removed
Self-assessed position in school at school class above median	Ref.	1.00	1.00	1.00	
Self-assessed position in school at school class below median		1.49 (1.12-1.99)	1.46 (1.08-1.98)	1.18 (0.86-1.62)	
Parental relations					
*Over-protection*					
Parents less over-protective than median	Ref.	1.00	1.00	1.00	1.00
Parents more over-protective than median		1.70 (1.29-2.25)	1.52 (1.12-2.06)	1.41 (1.03-1.92)	1.46 (1.08-1.95)
*Family Functioning*					
Family functioning above median	Ref.	1.00	1.00	1.00	removed
Family Functioning below median		1.51 (1.13-2.01)	1.25 (0.92-1.70)	1.14 (0.83-1.56)	
Personal psychological characteristics					
Self-esteem					
Self-esteem above median	Ref.	1.00	1.00	1.00	1.00
Self-esteem below median		1.66 (1.23-2.22)	1.51 (1.10-2.06)	1.33 (0.97-1.84)	1.41 (1.03-1.94)
Sense of coherence (meaningfulness)					
Sense of coherence above median	Ref.	1.00	1.00	1.00	removed
Sense of coherence below median		1.55 (1.17-2.05)	1.38 (1.02-1.87)	1.25 (0.92-1.70)	
Active coping					
Active coping above median		1.00	1.00	1.00	removed
Active coping below median		1.17 (0.88-1.55)	0.95 (0.70-1.29)	0.94 (0.69-1.28)	
Avoidance coping					
Avoidance coping below median	Ref.	1.00	1.00	1.00	removed
Avoidance coping above median		1.39 (1.04-1.84)	1.27 (0.95-1.71)	1.20 (0.89-1.63)	
Socioeconomic status					
Household income 2003					removed
Lowest tertile		1.45 (1.03-2.03)	1.32 (0.92-1.89)	1.18 (0.80-1.74)	
Middle tertile		1.40 (1.03-1.90)	1.38 (1.01-1.90)	1.25 (0.90-1.76)	
Highest tertile	Ref	1.00	1.00	1.00	
Household highest attained education 2003					removed
<10 year		1.65 (1.09-2.49)	1.47 (0.94-2.29)	*1.50 (0.92-2.43)*	
10-12 years		1.03 (0.77-1.37)	0.95 (0.71-1.28)	0.93 (0.68-1.27)	
>13 years	Ref	1.00	1.00	1.00	
Being bullied at age 14–15					
Not being bullied at age 14–15	Ref			1.00	1.00
Being bullied at age 14–15					3.14 (2.34-4.23)
Hosmer-Lemeshow Goodness-of-fit					Chi-square 23.44 = 0.02)
Nagelkerke Pseudo R^2^					0.06

^a^Adjusted for other factors in the same domain

**Adjusted for all variables with a p-value < 0.20

Finally, Table 5 shows the associations between the independent variables and experiencing bullying at the workplace at age 17–18. The strongest association observed for bullying at work was gender; boys had a more than twofold higher risk of experiencing bullying in the adjusted analysis. Having experienced bullying at school at age 14–15 raised the risk of being bullied at work, and this was also seen for bullying at secondary school. The association, however, was somewhat lower than the association between bullying at primary and secondary school. Parental relations also predict experiencing bullying: having more troublesome relationships with parents characterised by conflict and lack of communication raised the risk of bullying at work. And finally, scoring below the median on the meaningfulness dimension of the sense of coherence scale increased the risk of being bullied at work even after taking into account the experience of bullying at age 14–15. As was the case with the risk factors for bullying in school, several variables were bivariately associated with experiencing bullying at work at age 17–18. The effects of Body Mass Index, daily smoking and measures of social position in the peer group were all diluted after mutually adjusted for each other and for the experience of bullying at age 14–15. There was still, however, a slightly elevated risk for obese adolescents to experience bullying at work, even if the estimate was somewhat fragile.

**Table 5:** Associations between and lifestyle, personal characteristics, peer group and parental relations at age 15 and being bullied at work age 18. Odds ratios (OR) obtained by logistic regression with 95 % confidence intervals

		Model 1 Bivariate	Model 2^a^Adjusted for covariates in same domain	Model 3^a^Adjusted for covariates in same domain and bullying	Model 4** Forward stepwise selection
		OR (95 % CI)	OR (95 % CI)	OR (95 % CI)	OR (95 % CI)
Gender					
Girls	Ref	1.00		1.00	1.00
Boys		2.39 (1.68-3.41)		2.11 (1.44-3.09)	2.23 (1.48-3.35)
Lifestyle factors					
Body Mass Index					
Underweight		1.05 (0.32-3.47)	1.07 (0.32-3.55)	0.95 (0.28-3.23)	0.84 (0.24-2.95)
Normal weight	Ref	1.00	1.00	1.00	1.00
Overweight		1.95 (1.17-3.23)	1.94 (1.17-3.23)	1.84 (1.10-3.09)	1.77 (1.04-3.02)
Obesity		2.04 (0.84-4.96)	2.14 (0.88-5.22)	1.76 (0.71-4.35)	1.43 (0.56-3.64)
Smoking					
Does not smoke	Ref	1.00	1.00	1.00	1.00
Smoke		1.98 (1.02-3.85)	2.16 (1.10-4.24)	2.23 (1.12-4.43)	1.97 (0.94-4.11)
Social position in peer group					
School performance					
School performance above median	Ref.	1.00	1.00	1.00	removed
School performance below median		1.33 (0.91-1.94)	1.11 (0.74-1.68)	1.08 (0.71-1.64)	
Self-assessed position in school class					removed
Self-assessed position in school class above median	Ref.	1.00	1.00	1.00	
Self-assessed position in school class below median		1.84 (1.24-2.73)	1.78 (1.18-2.68)	1.49 (0.98-2.26)	
Parental relations					
Over-protection					
Parents less over-protective than median	Ref.	1.00	1.00	1.00	removed
Parents more over-protective than median		1.40 (0.97-2.02)	1.11 (0.75-1.64)	1.03 (0.69-1.54)	
Family Functioning					
Family functioning above median	Ref.	1.00	1.00	1.00	removed
Family Functioning below median		2.03 (1.37-3.01)	1.95 (1.29-2.95)	1.87 (1.23-2.85)	1.69 (1.10-2.59)
Personal psychological characteristics					
Self-esteem					
Self-esteem above median	Ref.	1.00	1.00	1.00	1.00
Self-esteem below median		1.65 (1.11-2.45)	1.37 (0.90-2.10)	1.29 (0.84-2.00)	1.60 (1.03-2.49)
Sense of coherence (meaningfulness)					
Sense of coherence above median	Ref.	1.00	1.00	1.00	removed
Sense of coherence below median		1.99 (1.36-2.91)	1.70 (1.13-2.57)	1.56 (1.03-2.37)	
Active coping					
Active coping above median	Ref.	1.00	1.00	1.00	removed
Active coping below median		1.40 (0.96-2.05)	1.13 (0.75-1.71)	1.09 (0.72-1.64)	
Avoidance coping					
Avoidance coping below median	Ref.	1.00	1.00	1.00	removed
Avoidance coping above median		1.32 (0.90-1.92)	1.17 (0.79-1.74)	1.14 (0.76-1.69)	
Socioeconomic status					
Household income 2003					
Lowest tertile		2.06 (1.29-3.28)	1.89 (1.15-3.11)	1.64 (0.96-2.80)	1.54 (0.89-2.67)
Middle tertile		2.17 (1.41-3.33)	2.16 (1.39-3.36)	1.95 (1.22-3.10)	2.10 (1.30-3.39)
Highest tertile	Ref.	1.00	1.00	1.00	1.00
Household highest attained education 2003					removed
<10 year		1.83 (1.07-3.11)	1.44 (0.81-2.55)	1.39 (0.73-2.65)	
10-12 years		1.13 (0.77-1.66)	0.94 (0.63-1.40)	1.01 (0.66-1.55)	
>13 years	Ref.	1.00	1.00	1.00	
Being bullied at age 14–15					
Not being bullied at age 14–15					1.00
Being bullied at age 14–15					2.09 (1.39-3.14)
Hosmer-Lemeshow Goodness-of-fit					Chi-square 166.26 = 0.48)
Nagelkerke Pseudo R^2^					0.09

^a^Adjusted for other factors in the same domain

** Adjusted for all variables with a *p*-value < 0.20

## Discussion

The first purpose of the present study was to examine the prevalence of being bullied at age 14–15 and age 17–18 at work and school. We found that that nearly 10 % of the participants reported being frequently bullied at age 14–15 during the last 6 months. This is in line with other studies. At age 17–18, the prevalence had decreased, with 4.7 % being frequently bullied at school and 5.5 % being bullied at work. This is also in accordance with previous studies, which have documented a decrease in the frequency of bullying during school life [15, 17]. Furthermore, at age 17–18 bullying was more frequent at work than at school.

The wording of the questions in 2007 was somewhat stricter, which meant that it was not possible to directly compare the prevalence of bullying at school between the two rounds.

The second purpose of the study was to identify the most important risk factors for being bullied at age 18, including risk factors at several levels. As mentioned earlier, aggressive behaviour such as bullying stems from the interplay of a wide range of personal, situational and social factors. In model 2, in which we mutually adjusted for other risk factors, we found that obesity, low self-assessed position in school class, overprotective parents, low self-esteem, low sense of coherence and middle socioeconomic status were all significant risk factors for being bullied 3 years later at school. In this same model (model 2), nearly identical risk factors (plus being a smoker) were identified at work. The results underline that bullying acts occur as a result of complex interactions between the persons involved and factors in the social context [12, 13], and furthermore, the results indicate somewhat similar risk factors for bullying across organisational settings (i.e. school and workplace).

In the next section, we will discuss the most important risk factors.

We found that physical appearance was a risk factor for being bullied both in school and at work. Obesity was a risk factor for being bullied at school, and overweight was a risk factor for being bullied at work. After controlling for previously being bullied, the association between obesity and bullying at school disappeared, but overweight remained a risk factor for being bullied at work. Several studies have found that overweight is a risk factor for being bullied [20–22]. The possible link between overweight and bullying could be that overweight adolescents have poorer psychological well-being and more depressive feelings, which would put them at increased risk of being bullied and furthermore affect their perceptions of other people’s teasing [65, 66]. Thus, it could be that it is the psychological consequences of being overweight, not simply being overweight, that increase the risk of being bullied.

At work, overweight remained a risk factor for being bullied even after adjusting for covariates such as self-esteem and sense of coherence. At work at least, it is more likely to be the physical appearance of overweight that is the determining factor behind the observed association. One mechanism might be that overweight workers may have difficulties keeping up with the rapid tempo of unskilled piecework jobs and thus be at risk of being bullied. For instance, in unskilled piecework in the construction sector, if a crew member has difficulties keeping up the tempo, he or she is at risk of informal sanctions [67].

The results show that being a smoker at age 15 is a significant risk factor for being bullied at age 18, but only at work. This association remains significant even after controlling for being bullied at age 14–15. One explanation might be that being a smoker is associated with lower social and economic status [68], which is often found to be a risk factor for being bullied [47, 69]. Another plausible interpretation is that smoking is increasingly being frowned upon because legislation regulating smoking at workplaces was tightened in 2007 when the adolescents were 17–18 years old. This would lead to smoking behaviour being more and more at odds with the prevailing norms in society.

Social position in the peer group was found to be a risk factor for being bullied both at work and at school. Previous studies have demonstrated that there is an association between low social status and increased risk of bullying [47, 69], and the present study adds to the current knowledge that low social position is a significant risk factor for being bullied irrespective of whether the adolescents at age 17–18 are at school or at work. One explanation could be that perceived popularity reflects dominance, and therefore it may be easy for a popular child or adolescent to bully others who are low in popularity without fear of being sanctioned by peers [46]. Consequently, bullying could be one way to maintain high status. Most studies examining the importance of social position in peer groups in relation to bullying have used cross-sectional designs [46, 47], but this study adds to the existing literature by using a longitudinal design, making the results more robust. However, after adjustment for previously being bullied, the associations were weaker. Furthermore, low school performance was not shown to be a risk factor for bullying in our study.

Social position in society, measured as socioeconomic status, was also found to be a risk factor both at work and at school. The negative association was seen for both low income and low education among parents, but it was diluted after adjusting for previously being bullied.

Having parents with a parenting style classified as overprotective was found to be a risk factor for being bullied at school, and low family function was a risk factor for being bullied at work even after adjusting for being bullied at age 14–15. In other words, parenting style at age 14–15 increases the risk of being bullied 3 years later. Other studies have also demonstrated an association between parenting style and being bullied [10, 32], and parental overcontrol has been found to be a risk factor for bullying among adolescent girls. The mechanism could be that parental overcontrol and protection limits adolescents’ opportunities to interact with certain kinds of peers and prevents adolescents from developing a diverse set of social skills [70]. Another mechanism could be that children, through their experiences at home (e.g. observational learning [71]), learn and reproduce insufficient ways of solving conflicts, which they reproduce at school or at work with their peers. Thus, parenting style may either increase the risk of being bullied or protect children from being bullied.

Low self-esteem was found to be a risk factor for being bullied both at school and at work. This is in line with previous findings [29, 30]. In the present study, the association was weakened after adjusting for previously being bullied. The mechanism could be that being bullied damages self-esteem, which would make it harder to manage teasing and bullying from peers.

Low levels of a sense of coherence were also a risk factor for being bullied both at school and at work. People with a strong sense of coherence are more resistant to stress and may be more insensitive to stressors like bullying [42]. We found that a weak sense of coherence increased the risk of being bullied. This result is in accordance with a Danish study that found that employees subjected to violence had a weaker sense of coherence [45].

Based on the size of the odds ratio, the increased risk for being bullied seems to be embedded first and foremost in individual and personal characteristics (obesity, low self-esteem, overprotective parents, low sense of coherence) and to a lesser degree in social contexts (low self-assessed position, low/middle socioeconomic status). Most likely the risk factors for being bullied reinforce each other, and future research may address the interaction between risk factors. After adjusting for being bullied at baseline, most of the aforementioned associations became insignificant, but irrespective of statistical significance, the size of the associations (odd ratio) between risk factors and bullying remained moderately strong or strong in most cases.

The third purpose of the study was to examine the continuity of being bullied from age 15 to age 18. The results showed that the highest risk (besides gender) for being bullied at age 17–18 at secondary school or at work was associated with being bullied at age 15. Apparently, being bullied once significantly increased the risk for future exposure to bullying. This is similar to previous findings demonstrating that being a victim of bullying increases the risk of later victimisation [8, 16, 56], which suggests a continuity of being bullied. In this study, the highest risk factor for being bullied was previously being bullied, and the results remained stable after adjustment for other risk factors. This degree of individual consistency of being bullied in different environments and at different ages points to factors of continuity in the risk of victimisation. One explanation may be that early bullying affects the perception of the self and relations to others, or the answer may be in individual attributes such as temperament, self-esteem, the ability to form protective relationships and coping. Temperament is a stable characteristic of the individual [72], but coping and self-esteem also seem to be rather stable over time. The stability of the coping response in adolescence was studied by Kirchner *et al.* [73], who found that coping responses were quite stable over time, especially avoidance coping, which may explain the continuing risk of bullying. Similar results have been found by other researchers [74, 75]. Furthermore, Alsaker and Olweus [76] found that adolescents’ negative self-evaluation (self-esteem) is likely to become relatively more crystallised with increasing age. These findings suggest the importance of certain individual characteristics as risk factors for later bullying at school and at the workplace.

However, there is also a substantial degree of discontinuity in being bullied. Many victims of early school bullying do not become workplace victims or victims of bullying at secondary school. This suggests that although stable individual factors may have some importance in explaining victimisation risk, contextual factors such as immediate environment and social support are also important. This is supported by research that has shown that the most important risk factors for the development of bullying in the workplace on the team and organisational level were leadership style, norms and values, and communication and social climate [77]. Furthermore, employees that experienced bullying had lower perceptions of their work environment in general, especially in relation to trust, cooperation, conflict resolution and justice in the organisation [78]. Thus, a great deal of the variance in workplace bullying may be due to environmental factors.

### Limitations

Although the study reveals significant associations between risk factors and bullying, caution about causal inference is warranted. Firstly, it can be argued that a 3-year gap between exposure to bullying and the measurement of bullying is too long. However, a certain amount of time between baseline and follow-up data is necessary as one main criterion of the definition of being bullied is the prolonged nature of the negative experience [2], and negative acts often develop over a long time span [79]. In addition, it is unknown to what extent the associations reported in this paper are weakened due to dropouts among those being bullied at age 14–15 from the first to the second questionnaires, but the response rate was still 59 % of the original cohort.

Furthermore, the results should be interpreted with some caution because the study is based only on self-reported measures, which increases the risk of common methods bias [80]. Furthermore, it is also worth noting that the measurement of bullying consisted of a single item, leaving it up the individual participant to define the concept of bullying. However, self-reported measures are common in this area of research and seem to yield reliable and valid results [81].

Finally, the data did not contain information on personality traits such as neuroticism that could affect the reporting of bullying and the predictor variable.

### Future research

The results stress the importance of more prospective cohort studies. The underlying and longitudinal psychosocial mechanisms of being bullied are unclear and may be mediated through a decreased ability to adapt sufficiently. More prospective studies with several time lags are needed to determine the moderating and mediating psychological processes between risk factors and bullying and to determine whether the increased risk of early victimisation from being bullied remains a risk factor into adulthood. Furthermore, future research must focus on factors related to the individual or family context that can moderate negative factors and make the individual more resilient. Finally, future research must examine the importance of personality. So far the results concerning personality as a risk factor are inconsistent, and there is a need for more prospective studies to determine whether neuroticism predisposes children to victimisation in school or whether victimisation affects the development of a vulnerable personality [82]. In addition, there is a need for qualitative studies exploring in detail the psychological processes leading from being bullied once to an increased vulnerability to later victimisation.

Finally, the discontinuity in being bullied may be a focus for future research because many victims of early school bullying do not become workplace victims or victims of bullying at secondary school. It is importance to identify protective factors at both the personal and organisational levels.

Another important addition to our paper would have been the implementation of more sophisticated statistical modelling, such as structural equation modelling, e.g. using cross-lagged models. This would have made it possible to gain more knowledge about the associations between the risk factors examined in this paper and the two outcomes: bullying at work and bullying at school. This type of analysis has the potential to better illuminate the possible causal associations between the risk factors for bullying identified in this study. However, we were not able to use cross-lagged modelling of bullying for two reasons. First of all, in the first round of the questionnaire there was only one question on bullying (because the adolescents were too young (14–15) to have a job in which they spent enough time to allow for an exposure to bullying). Secondly, the wording of the questions was slightly different, which also prohibit a proper cross-lagged analysis. Finally, if the aim was to cross-lag socioeconomic status and bullying, this would also be difficult because the measure of socioeconomic status is of the educational level and income in the household in which the adolescents are living. This means that those adolescents that have moved out of their parent’s homes would no longer have a comparable socioeconomic status measure, which again makes the cross-lagged approach very difficult because of the transitional phase the adolescents were in at the time of participation in the study.

## Conclusion

The results show that the most important risk factors for being bullied at age 17–18, whether at work or in school, were being a boy and previously being bullied. This result stresses the importance of early prevention of bullying at schools. In addition, having overprotective parents was a risk factor for being bullied at school. Being overweight, smoking, experiencing low family function and having low socioeconomic status were additional risk factors for being bullied at work.

In summary, the increased risk for being bullied seems to be embedded first and foremost in individual and personal characteristics, to a lesser degree in the social context, but most importantly, in the experience of previously being bullied.
